# Beyond the Binary: A Five-Pillar Framework for Hybrid Medical Education Post-Pandemic

**DOI:** 10.1177/23821205251395293

**Published:** 2025-11-14

**Authors:** James R. Burmeister, Ismail Zazay, Rebecca L. Pratt

**Affiliations:** 1 Department of Foundational Medical Studies, 159878 Oakland University William Beaumont School of Medicine, Rochester, MI, USA; 2 John Sealy School of Medicine, 12338 University of Texas Medical Branch, Galveston, TX, USA

**Keywords:** Hybrid education, medical curriculum, post-pandemic teaching, clinical readiness, instructional design, equity in education

## Abstract

The COVID-19 pandemic rapidly transformed medical education, exposing both challenges and opportunities in online, in-person, and hybrid instructional models. This Viewpoint proposes a five-pillar framework for effective hybrid medical education: curricular alignment, faculty development, interactive online pedagogy, flexibility with accountability, and data-driven feedback. Drawing on the experience of two medical students and the perspective of an anatomy professor, an award-winning educator and curriculum leader, we offer a framework for hybrid medical education. While our viewpoint centers on undergraduate medical education, we recognize concerns from graduate medical educators about downstream resident preparedness and the need for broader collaboration. Our framework addresses learner engagement, clinical preparedness, and equity. We recommend that academic leaders adopt and periodically audit curricula using this framework to ensure educational quality and readiness for future disruptions.

In the wake of COVID-19, medical education underwent a rapid transformation as institutions adopted online and hybrid teaching out of necessity, often in ways that were hurried and lacked rigorous prior evaluation. While these adaptations allowed educational continuity, most changes were implemented without robust outcome data, and their comparative effectiveness remains uncertain.^1,2^ To date, the literature offers limited and sometimes conflicting evidence regarding the benefits of post-pandemic curricula compared to traditional models.^1,2^

As we return to more stable instructional modes, the goal should not be to revert to past norms or to uncritically embrace online delivery, but rather to design an intentional, evidence-informed integration of modalities, subject to ongoing evaluation and improvement. Our proposed five-pillar framework is intended as a starting point for curriculum development and research, not as a definitive solution.

Studies in health professions education show that flipped-classroom approaches significantly improve motivation, critical thinking, and peer collaboration.^3,4^ For example, Ayoub and Abd El-Aziz (2024) demonstrated that medical students in flipped-classroom settings reported higher engagement and achieved greater academic success than those in traditional lectures.^3^ Similarly, Hew and Lo (2018) found in their meta-analysis that the flipped classroom model led to improved learning outcomes across various health professions.^4^

In addition, recent studies indicate that generative Artificial Intelligence (AI) tools can significantly improve pharmacology retention and student participation by providing interactive, adaptive, and engaging learning experiences.^5^ Boddepalli and Solanke (2025) reported that medical students using AI platforms had better retention and were more motivated to participate in self-directed learning activities.^5^

On the other hand, research demonstrates that passive, recorded lectures, especially when lacking synchronous or interactive components, are associated with reduced student attendance and attention, underscoring the limitations of unstructured online content.^1,2^ True online learning must transcend the emergency model of long, recorded lectures. Instead, concise, objective-driven modules paired with live, expert-led discussions, peer teaching, and active clinical applications ensure engagement and promote long-term retention.

Second, clinical preparedness remains a critical concern. While online standardized-patient encounters and telehealth simulations gained popularity during the pandemic, students in fully online clerkships reported reduced confidence in bedside exams and physical diagnosis.^6,7^ In contrast, hybrid and simulation-based curricula that combine online and in-person components have shown promise in preserving clinical skill competence.^6,7^ Anatomy education exemplifies this balance: cadaver-based dissection offers irreplaceable tactile and spatial experiences, while online tools can reinforce understanding.^8^ Furthermore, in-person group discussions and oral case analyses generate real-time feedback for learners and faculty alike. These moments foster oral communication, critical thinking, and retrieval-based learning, elements that are central to clinical readiness.^9^ Importantly, in-person group learning is also foundational for professional identity formation, professional socialization, and the development of communities of practice within medicine.^9,10^ These processes, which occur most robustly through shared dialogue and mentorship, are vital for shaping the values, norms, and professional behaviors of future physicians, yet were significantly disrupted or abandoned during the COVID-19 era.

Our five-pillar framework is strengthened by explicitly recognizing the importance of these social and developmental dimensions. As medical education continues to evolve, hybrid and online models must intentionally reintegrate opportunities for authentic professional socialization and community-building, not just knowledge transfer. Relying solely on multiple-choice exams or asynchronous content undermines these essential aspects of physician development.

Third, teaching effectiveness hinges on faculty development and institutional support. Many faculty adapted impressively to remote teaching, but others experienced burnout and pedagogical stagnation.^9^ As one of our faculty members reflects:

Transitioning to hybrid teaching required not only new technical skills, but also a complete redesign of how I connect with students and provide timely feedback. Institutional support in the form of collaborative training and protected time was essential to maintaining teaching quality.

Institutions must support faculty with more than technical training. Professional development should include instructional design for hybrid environments, online assessment strategies, and fostering interaction across modalities. However, such large-scale curricular transformation should not rest solely on the initiative or volunteerism of individual faculty members, who are often under-resourced and lack protected time.

National leadership bodies such as the Liaison Committee on Medical Education (LCME) and Association of American Medical Colleges (AAMC) must play a central role in guiding, standardizing, and resourcing hybrid curriculum redevelopment across institutions. Coordinated guidance and dedicated funding will be essential to ensure equitable, sustainable, and high-quality reform, allowing faculty to focus on effective teaching rather than administrative burden.

Crucially, institutions must establish standards for instructional quality aligned with learner engagement and readiness, not just student satisfaction. Faculty need protected time and robust institutional support to meet these evolving demands.

Fourth, well-being and equity must be integral to any curricular redesign. Longstanding issues of student burnout intensified during the pandemic.^11^ Moreover, many learners and educators face complex post-pandemic realities: caregiving responsibilities, chronic illness, or financial hardship may necessitate flexible learning options. Yet for others, poor internet access or unstable home environments make remote learning difficult.^12^ Institutions must invest in supportive infrastructure, technology access, mental health resources, and on-campus study spaces, while designing flexible, accountable systems that serve all learners fairly.

Fifth, we must shift from traditional contact hours to a greater emphasis on competency-based outcomes, as endorsed by national educational organizations. In competency-based medical education (CBME), the focus is on students’ demonstrated skills, knowledge, and professional behaviors, rather than time spent in a particular setting. While online and hybrid modalities can provide additional learning opportunities and flexibility, the current evidence does not yet establish equivalence to in-person education for all competencies, particularly those requiring direct observation and assessment.^12^

The Master Adaptive Learner model emphasizes the importance of preparing learners for lifelong learning and adaptability in complex, often unpredictable clinical environments.^12^ This approach seeks to equip future physicians with strategies for continuous growth and adaptation, recognizing that the healthcare workplace will not always provide optimal learner-centered or flexible options. Therefore, while incorporating hybrid methods may offer advantages, medical education must ensure that graduates are prepared to thrive across diverse practice settings, including those with less flexibility or more traditional demands.

Breaking free from rigid, time-based frameworks can empower medical schools to prioritize mastery, but this transition must be grounded in careful, ongoing evaluation of educational outcomes, with close attention to the skills and professional attributes required for clinical readiness and patient care.

Our framework reflects the perspectives of undergraduate medical students and a faculty member engaged in curriculum delivery. In this viewpoint, we did not directly include the views of graduate medical education (GME) program directors or residency leadership. Notably, recent literature and residency program directors have raised concerns that increasing flexibility in medical school may affect graduates’ adjustment to the demands of residency, with possible gaps in clinical and professional readiness. Recent findings by Grbic et al. (2025) indicate that medical graduates whose training was disrupted by the COVID-19 pandemic were less likely to meet residency program expectations in their first six months, underscoring the complexity of preparedness and the importance of ongoing collaboration between undergraduate medical education (UME) and GME educators across all specialties.^13^ Therefore, future curriculum reform and research should actively involve GME stakeholders to ensure alignment across the training continuum.

While a learner-centered approach can enhance engagement and flexibility, we recognize that the overarching objective of UME must remain the development of clinically prepared, professionally responsible physicians. This includes fostering the ability to prioritize patient needs above self-interest, as emphasized in the Accreditation Council for Graduate Medical Education (ACGME) Common Program Requirements.

In sum, we propose the following five pillars to guide hybrid curricular reform:

1. Instructional Alignment: Match content to the most effective modality. Use in-person instruction for procedural, team-based, or spatially complex learning; leverage online formats for foundational knowledge paired with interactive strategies.
2. Interactive Online Pedagogy: Design online content with active learning in mind. Use quizzes, discussions, generative AI, and flipped models to engage learners.
3. Faculty Development: Equip educators with skills for hybrid instruction, including design, assessment, and maintaining pedagogical standards.
4. Flexibility with Accountability: Support students and faculty through life transitions, while ensuring robust participation tracking and feedback systems.
5. Data-Driven Feedback Loops: Use objective data, including performance on knowledge assessments, observed skills development, and clinical readiness milestones, in addition to engagement metrics and learner input, to iteratively improve curriculum. Feedback mechanisms should prioritize evidence of learning and competence over learner satisfaction alone.

Given the observed decrease in United States Medical Licensing Examination (USMLE) Step 1 pass rates and ongoing concerns from residency program directors, further research and careful evaluation of hybrid and flexible curricula are critical before widespread adoption. This concern is supported by recent USMLE Step 1 pass rate data and aligns with earlier findings by Hall et al, who documented residency director concerns about preparedness even prior to the pandemic.^14^ Our recommendations should be viewed as a starting framework for discussion and pilot testing, rather than a universal solution.

Medical education stands at a curricular crossroads. The pandemic dismantled assumptions about where and how learning should occur. Rather than retreat to tradition or default to digitization, we must construct a new model centered on competency, professional development, and equity, while thoughtfully incorporating flexibility as one important component. The goal of UME should be to prepare students for successful transitions to residency, fellowship, and independent practice, with deliberate attention to the development of the knowledge, skills, professional identity, and socialization required for lifelong success in medicine.

By adopting this five-pillar framework, academic medicine can foster an educational ecosystem that not only supports learner engagement but, more importantly, prioritizes clinical readiness, professional formation, and the cultivation of communities of practice that safeguard both the public and the profession.
